# Ahnak is required to balance calcium ion homeostasis and smooth muscle development in the urinary system

**DOI:** 10.1186/s13578-023-01055-x

**Published:** 2023-06-12

**Authors:** Jong-Min Lee, Tae-Yang Lim, Sang-Bin Oh, Seung-Jun Lee, Yun Soo Bae, Han-Sung Jung

**Affiliations:** 1 Department of Oral Biology, Taste Research Center, Oral Science Research Center, BK21 FOUR Project, Yonsei University College of Dentistry, Seoul, 03722 Korea; 2 Department of Life Sciences, Ewha Woman’s University, Seoul, Korea

**Keywords:** Ahnak, Kidney, Cellular calcium ion homeostasis, Hydronephrosis, Ureter, Muscle development, Hydroureter

## Abstract

**Background:**

Various renal abnormalities, including hydronephrosis, polycystic kidney disease, and hydroureter, have been reported, and these abnormalities are present in DiGeorge syndrome, renal dysplasia, and acute kidney failure. Previous studies have shown that various genes are associated with renal abnormalities. However, the major target genes of nonobstructive hydronephrosis have not yet been elucidated.

**Results:**

We examined neuroblast differentiation-associated protein Ahnak localization and analyzed morphogenesis in developing kidney and ureter. To investigated function of Ahnak, RNA-sequencing and calcium imaging were performed in wild type and Ahnak knockout (KO) mice. Ahnak localization was confirmed in the developing mouse kidneys and ureter. An imbalance of calcium homeostasis and hydronephrosis, which involves an expanded renal pelvis and hydroureter, was observed in Ahnak KO mice. Gene Ontology enrichment analysis on RNA-seq results indicated that ‘Channel Activity’, ‘Passive Transmembrane Transporter Activity’ and ‘Cellular Calcium Ion Homeostasis’ were downregulated in Ahnak KO kidney. ‘Muscle Tissue Development’, ‘Muscle Contraction’, and ‘Cellular Calcium Ion Homeostasis’ were downregulated in Ahnak KO ureter. Moreover, peristaltic movement of smooth muscle in the ureter was reduced in Ahnak KO mice.

**Conclusions:**

Abnormal calcium homeostasis causes renal disease and is regulated by calcium channels. In this study, we focused on Ahnak, which regulates calcium homeostasis in several organs. Our results indicate that Ahnak plays a pivotal role in kidney and ureter development, and in maintaining the function of the urinary system.

**Supplementary Information:**

The online version contains supplementary material available at 10.1186/s13578-023-01055-x.

## Background

A complex urinary system produces urine to maintain body homeostasis by directing the amount of bodily fluids, electrolyte balance, and excretion of metabolic end products [1, 2]. Several clinical disorders of the urinary system are organized by congenital anomalies of the kidney and urinary tract (CAKUT) [3]. CAKUT shows abnormalities in the kidneys or other structures of the urinary tract, including underdevelopment or absence of a kidney (renal hypodysplasia or agenesis), abnormally wide kidney pelvis/calyxes, and a ureter lumen (hydronephrosis and hydroureter) [4, 5].

Obstruction or dilation of the urinary tract is observed in patients with hydronephrosis [6, 7]. Hydronephrosis is categorized into grades 0–4. No dilation is observed in grade 0. Grade 1, pelvic dilation only; Grade 2, mild caliceal dilatation; Grade 3, severe caliceal dilation; Grade 4, renal parenchymal atrophy [8].

Non-obstructive hydronephrosis, resulting in kidney failure and deformation, is caused by improper filtration of excessive amounts of urine [9, 10]. Persistent urine volume overload disrupts urine flow and results in reflux [8]. Previous studies have reported that disruption of calcium homeostasis in the kidney induces non-obstructive hydronephrosis by urine overload [11]. Non-obstructive hydronephrosis patients exhibit abnormal peristalsis movement involving the dilated ureter [12]. In addition, acute kidney injury can cause chronic kidney disease and the kidney is an organ that has limitations for repairing itself [13]. For this reason, understanding kidney disease-associated genes is important for preventing acute kidney diseases. However, the genetic causes of nonobstructive hydronephrosis have not been clearly elucidated.

Since the identification of neuroblast differentiation-associated protein Ahnak in skin keratinocytes and neuroblastoma cells, many reports have indicated that Ahnak serves a scaffolding function in various cell signaling cascades [14]. Ahnak is expressed in several intracellular locations, including the plasma membrane, cytoplasm, and nucleus [15]. The carboxyl-terminal region of Ahnak proteins has been reported to play important roles in cellular localization and in interaction with L-type Ca^2+^ channels in cardiac cells [16]. A previous study employing single cell RNA-sequencing revealed that Ahnak is expressed in mature podocyte clusters [17]. However, the underlying molecular mechanisms of Ahnak in the kidney and ureter are poorly understood.

To confirm the function of Ahnak in the kidney and ureter, we performed an analysis of Ahnak knockout (KO) mouse kidneys and ureters. Hydronephrosis was observed in Ahnak KO mouse kidneys, and obstruction was not observed in the distal ureter of Ahnak KO mice. Bulk RNA sequencing was performed to investigate the genetic regulation of Ahnak. We found that Ahnak KO mice had negatively regulated genes for kidney development, calcium homeostasis, and ureteric smooth muscle differentiation. These results indicate that Ahnak may contribute to urinary system development, smooth muscle development, and the maintenance of calcium homeostasis.

## Results

### Localization of Ahnak in developing kidney and ureter

**Fig. 1:**
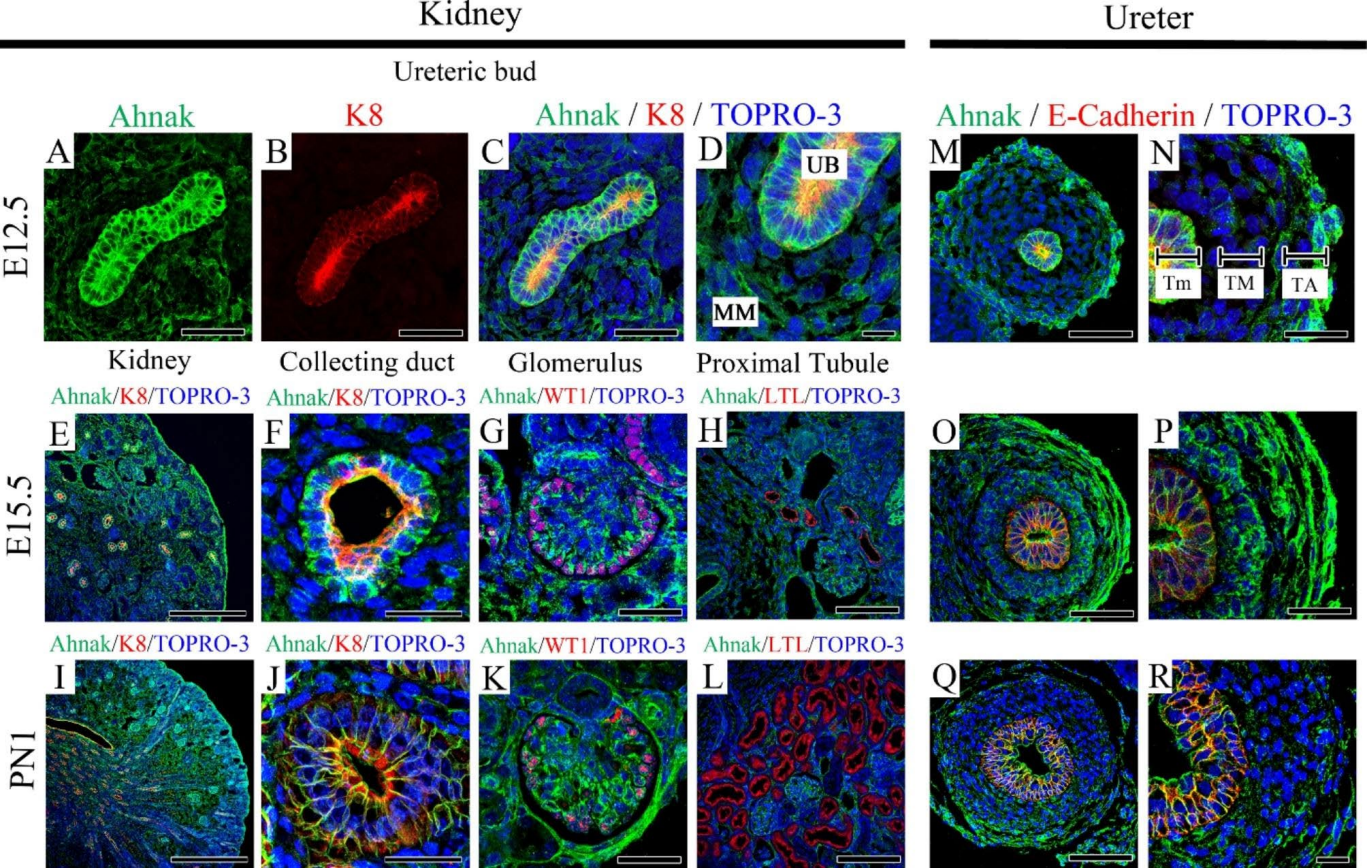
Localization of Ahnak in developing WT kidney and ureter. (**A-D**) Ahnak is localized in ureteric bud (UB) at E12.5 WT kidney. K8 marks collecting duct, co-localized with Ahnak in the collecting duct cell membrane. (**D**) High magnification image showing that Ahnak is strongly localized in the UB but weakly in the metanephric mesenchyme (MM). (**E**) At E15.5 WT kidney, Ahnak is detected in collecting duct and glomerulus. Renal mesenchyme has weak Ahnak localization. (**F**) Ahnak is localized in collecting duct membranes with K8. (**G**) In glomerulus, Ahnak is detected in podocytes. (**H**) Double staining results shows that Ahnak is not co-localized with lotus tetragonolobus lectin (LTL), a proximal tubule marker. (**I-L**). The overall localization tendency of Ahnak at PN1 is similar to E15.5. (**M-R**) Ahnak and E-cadherin are co-localized in tunica mucosa. Ahnak is detected in tunica muscularis and tunica adventitia during ureter development. UB; Ureteric bud, MM; Metanephric mesenchyme, Tm; Tunica mucosa TM; Tunica muscularis TA; Tunica adventitia. Scale bar; A, B, C, G, K, N, P, R, 100 μm; D, 150 μm; E, 250 μm; F, J, 40 μm; H, M, O, Q, 100 μm; I, 500 μm; L, 200 μm

Localization of Ahnak in the developing kidney and ureter was examined using immunohistochemistry (IHC). At embryonic day (E) 12.5, Ahnak was strongly localized in the ureteric bud (UB) and less so in the underlying metanephric mesenchyme (MM) (Fig. 1. A-D). Ahnak co-localized with K8, a collecting duct marker, in the kidney at E12.5. At E15.5, Ahnak was broadly localized in the kidney (Fig. 1E). Ahnak colocalized with Keratin-8 (K8) in the collecting duct (Fig. 1F). In the glomerulus, the podocyte-specific marker Wilms tumor-1 (WT1) was co-localized with Ahnak at E15.5 (Fig. 1G). Ahnak was not co-localized with Lotus Tetragonolobus Lectin (LTL) in the proximal tubule of the kidney at E15.5 (Fig. 1H). At PN1, Ahnak was similarly localized in the collecting duct, glomerulus, and proximal tubule in the kidney compared to E15.5 (Fig. 1I-L). Low magnification imaging indicated that Ahnak was broadly detected in the kidneys at PN1 (Fig. 1I). Ahnak was detected in the collecting duct co-localized with K8 and glomerulus with WT1, but not in the proximal tubule at PN1 in the kidney (Fig. 1J-L).

At E12.5, Ahnak was strongly localized in the urothelium and tunica adventitia (TA) layer but weakly localized in the tunica muscularis (TM) (Fig. 1M). High magnification imaging indicated that Ahnak was strongly detected in the tunica mucosa (Tm) layer colocalized with E-cadherin (Fig. 1N). At E15.5, Ahnak was strongly localized in three layers of the developing ureter (Fig. 1O). High magnification imaging showed that Ahnak was strongly localized in the Tm, TM, and TA at E15.5 in the ureter (Fig. 1P). At PN1, Ahnak was strongly detected in the Tm and weakly detected in the TM and TA in the ureter (Fig. 1Q). High magnification imaging indicated that Ahnak was strongly localized in Tm with E-cadherin, but weakly localized in TM and TA in the PN1 ureter (Fig. 1R).

### Morphological differences between WT and ahnak KO mouse kidney and ureter

**Fig. 2:**
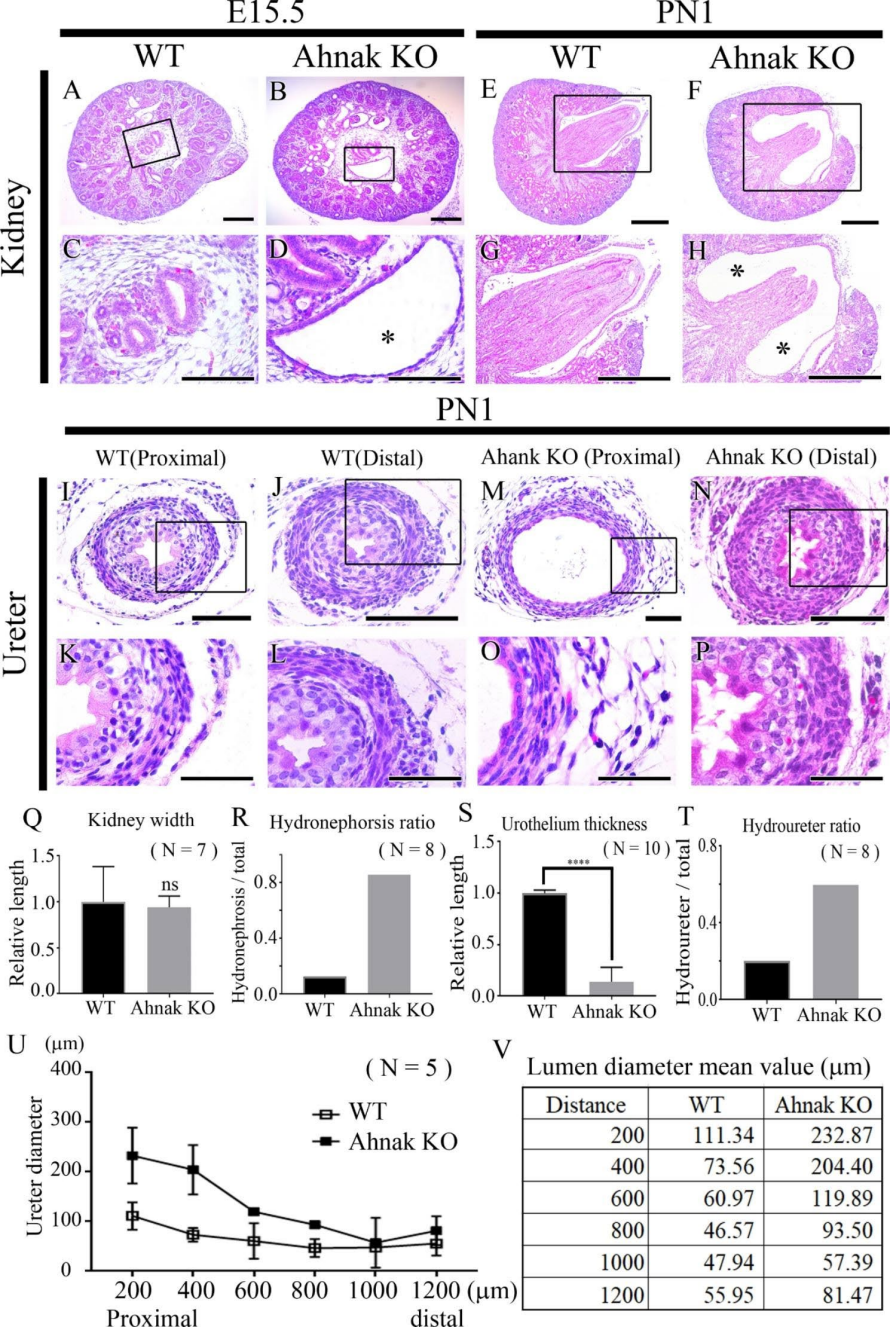
Phenotypic comparison between WT and Ahnak KO kidney and ureter. (A-H) At E15.5 and PN1, hydronephrosis with expanded vacant region (asterisks) is observed in Ahnak KO kidney compared to WT. (**I-P**) Well developed tunica mucosa, tunica muscularis and tunica adventitia are observed in WT and Ahnak KO distal ureter. Abnormal tunica mucosa, tunica muscularis and tunica adventitia formation is detected in Ahnak KO proximal ureter. (**Q**) Kidney width is not significantly different between WT and Ahnak KO mice. (**R**) Hydronephrosis ratio is increased approximately 8-fold in Ahnak KO kidney. (**S**) Urothelium thickness is significantly reduced in Ahnak KO ureter compared with WT. (**T**) Hydroureter ratio is dramatically increased in Ahnak KO ureter. (**U, V**) Relative lumen diameter per 200 μm position from proximal to distal region shows that hydroureter presents proximal region in Ahnak KO at PN1. Scale bar; A, B, C, D, 100 μm; E, F, G, H, 500 μm; I, J, M, N, 100 μm; K, L, O, P 50 μm

To investigate the phenotype of Ahnak KO mouse kidneys, we performed hematoxylin and eosin (H&E) staining at E15.5 and PN1. H&E staining results indicated that hydronephrosis was observed in Ahnak KO mouse kidneys at E15.5, compared to wild-type (WT) (Fig. 2A-D). Hydronephrosis was also detected at PN1 in Ahnak KO kidneys, similar to E15.5 (Fig. 2E-H). The kidney width was not significantly different between WT and Ahnak KO kidneys (Fig. 2Q). However, the hydronephrosis ratio was higher in Ahnak KO kidneys than in WT kidneys (Fig. 2R).

Morphological differences between WT and Ahnak KO ureters were confirmed by H&E staining at PN1 (Fig. 2I-P). The lumen of the ureter, Tm, TM, and TA was well developed in both the WT proximal and distal ureters at PN1 (Fig. 2I-L). A hydroureter was observed in the Ahnak KO proximal ureter at PN1 (Fig. 2M, O). Ahnak KO ureters showed mono- or double-layer Tm (Fig. 2O) compared to the multilayer urothelium in the WT ureter (Fig. 2K). Significant morphological differences were not detected between Ahnak KO and WT distal ureters (Fig. 2J, L, N, and P).

The thickness of the urothelium, hydroureter ratio, and ureter diameter were analyzed in WT and Ahnak KO mice at PN1 (Fig. 2S-V). The urothelium thickness was reduced in Ahnak KO mice compared to that in WT mice (n = 10) (Fig. 2S). The hydroureter ratio was significantly higher in the proximal region of the ureter compared than in WT mice (n = 8) (Fig. 2T). The diameter of the ureter was measured every 200 μm from proximal to distal in WT and Ahnak-KO mice. Increased ureter diameter was observed in the proximal region of Ahnak KO ureters compared to that in WT ureters (Fig. 2U, V). However, a similar diameter was observed in the distal region of the WT and Ahnak KO ureters (n = 5).

### RNA-sequencing analysis in Ahnak knockout kidney and ureter

**Fig. 3:**
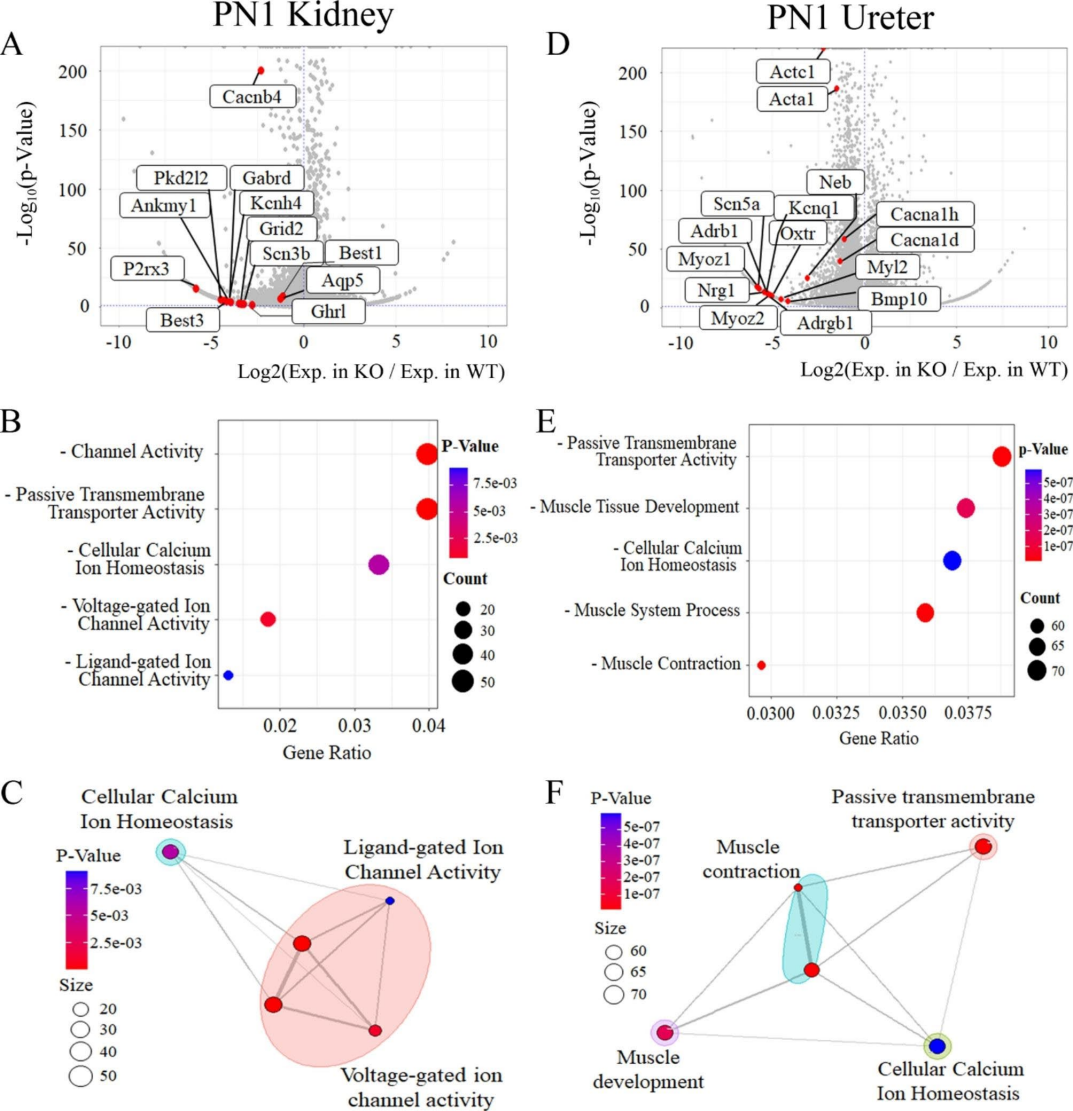
Bulk RNA-seq analysis of kidney and ureter in Ahnak KO and WT mice. (**A**) Volcano plot showing that expression levels of calcium ion homeostasis-related genes such as *Best3*, *Cacnb4* and *P2rx3* are reduced in Ahnak KO kidney compared to WT. (**B**) Analysis of gene ontology (GO) indicates that channel activity, passive transmembrane transporter activity, cellular calcium ion homeostasis, and ion channel activity-related GO terms are reduced in Ahnak KO kidney compared to the WT kidney. (**C**) EMAP plot showing the reduced calcium related functions and connections representing shared genes in Ahnak KO mouse kidney. (**D**) Volcano plot presenting down-regulated genes related to calcium homeostasis and muscle development, such as *Adrb1* and *Cacna1d* in Ahnak KO ureter. (**E**) Analyzed results of down-regulated GO terms show that passive transmembrane transporter activity, muscle tissue development, cellular calcium ion homeostasis, muscle system processes, and muscle contraction are reduced in Ahnak KO ureter compared to the WT. (**F**) EMAP plot showing that changed GO terms have a network each other and that all reduced GO terms are related to muscle development, contraction and calcium homeostasis

To identify gene expression differences between WT and Ahnak KO mouse kidneys, bulk RNA sequencing was performed. A volcano plot demonstrated that 1,467 genes (Log_2_FC > 0.5) were reduced, whereas 533 genes (Log_2_FC > 0.05) were induced in Ahnak KO kidneys compared with WT kidneys at postnatal (PN) 1 (Fig. 3A). Decreased genes expression of Ahnak KO was observed in the volcano plot compared with WT in the kidney. The top-12 downregulated differentially expressed genes (DEGs), including calcium signaling regulators such as *Cacnb4* (Calcium Voltage-Gated Channel Auxiliary Subunit Beta 4), *Best3 (*Bestrophin 3) and *P2rx3* (Purinergic Receptor P2 × 3), were presented in a volcano plot for Ahnak KO kidneys (Fig. 3A; DEGseq *P* < 0.05 and fold change > 2; a list of the DEGs is shown in Additional file 1: Table S2).

Gene Ontology (GO) enrichment analysis was performed on Ahnak KO kidneys. GO enrichment analysis results showed that genes associated with ‘Channel Activity’, ‘Passive Transmembrane Transporter Activity’ and ‘Cellular Calcium Ion Homeostasis’ were downregulated in Ahnak KO kidney (Fig. 3B). These results indicate that the expression of genes associated with renal calcium signaling was reduced in Ahnak KO kidneys. An EMAP plot showed gene set enrichment in the kidney (Fig. 3C). EMAP plot presentation indicated that ‘Cellular Calcium Iron Homeostasis’, ‘Ligand-gated Iron Channel Activity’, and ‘Voltage-gated Ion Channel Activity’ were downregulated in Ahnak KO kidneys at PN1.

Bulk RNA sequencing was performed to identify gene expression differences between WT and Ahnak KO mouse ureters. In Ahnak KO ureters, 2,115 genes (Log_2_FC > 1) were reduced, but 1,150 genes (Log_2_FC > 1) were induced at PN1 compared to WT (Fig. 3D). The volcano plot was labeled the top-15 downregulated DEGs in Ahnak KO ureter (Fig. 3D; DEGseq *P* < 0.05 and fold change > 2; list of DEGs shown in Additional file 1: Table S3) related to muscle development and contraction such as *Adrb1*, *Actc1*, *Bmp10*, *Myoz1*, and *Acta1* (Fig. 3D). In the ureter, the major significant genes, *Adrb1*, *Actc1*, *Bmp10*, *Myoz1*, and *Acta1*, were localized in related GO terms such as ‘Muscle Tissue Development’, ‘Muscle Contraction’, and ‘Cellular Calcium Ion Homeostasis’ (Fig. 3E). In addition, EMAP plot results indicated that linked ‘Muscle Development’, ‘Muscle Contraction’, ‘Passive Transmembrane Transporter Activity’ and ‘Cellular Calcium Ion Homeostasis’ related genes were down-regulated in Ahnak KO ureter compared to WT (Fig. 3F).

### Analysis of kidney abnormalities in Ahnak KO mouse at PN1

**Fig. 4:**
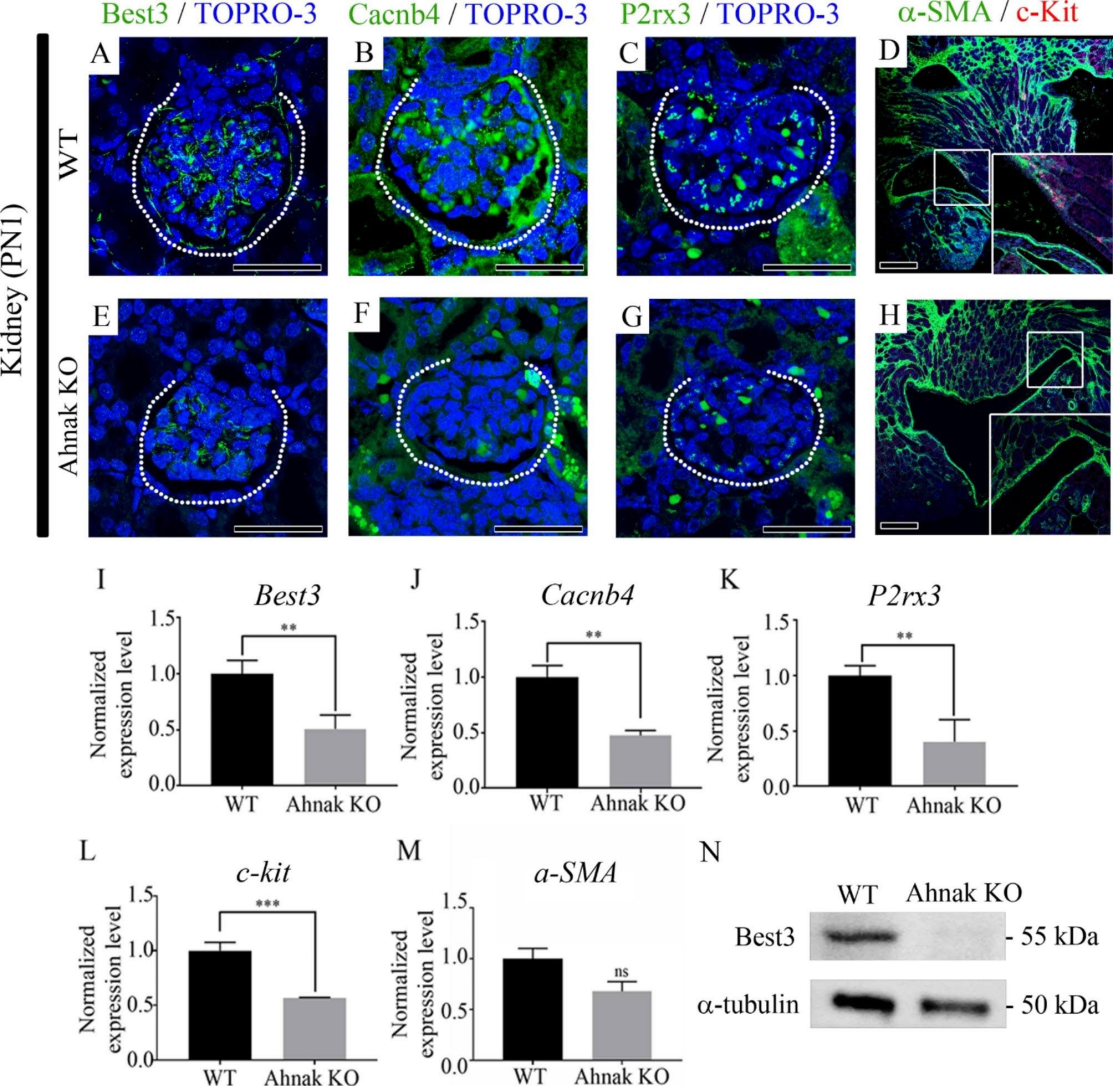
Calcium and peristalsis-related protein localization and gene expression in Ahnak KO mouse kidney at PN1. (**A**) Best3 is broadly observed in WT glomerulus. Best3 is localized in glomerulus and proximal tubules. (**E**) Best3 is weakly detected in Ahnak KO kidney compared with WT kidney. (**B**) Cacnb4 is strongly observed in glomerulus and surrounding tubules. (**F**) Cacnb4 is faintly detected in Ahnak KO kidney compared to WT. (**C**) P2rx3 is broadly and strongly localized in WT glomerulus and surrounding tubules. (**G**) P2rx3 is weakly observed in Ahnak KO kidney compared to WT. (**D**) Peristalsis marker c-kit is localized in basement membrane of WT kidney pelvis. (**H**) c-kit is not detected in Ahnak KO kidney pelvis. (**I**) Expression level of *Best3* is reduced in Ahnak KO kidney compared with WT at PN1. (**J**) *Cacnb4* expression level is down-regulated in Ahnak KO compared to WT. (**K**) Expression level of *P2rx3* is decreased in Ahnak KO compared with WT kidney at PN1. (**L**) Expression level of c-kit is reduced in Ahnak KO kidney. (**M**) Expression level of α-SMA is not altered in Ahnak KO kidney at PN1. (**N**) Best3 protein level was reduced in Ahnak KO mouse Kidney at PN1. White dotted line, glomerulus; **, *P* < 0.01; ***, *P* < 0.001; ns, not significant, Scale bar; A-H 100 μm

To investigate the reactivity of calcium ion channels in the kidney, IHC was performed using Best3, Cacnb4, and P2rx3 (Fig. 4A-C, E-G). Calcium channel markers, including Best3, Cacnb4, and P2rx3, were weakly or not detected in the glomerulus of Ahnak KO kidneys (Fig. 4E-G) compared with the glomerulus in WT at PN1 (Fig. 4A-C). c-kit, a peristalsis trigger protein, was localized in the pelvic epithelial layer of the medulla in the WT kidneys (Fig. 4D). However, c-kit was not localized in kidney pelvic epithelial layer of the medulla of Ahnak KO kidneys (Fig. 4H). Expression levels of calcium ion channels and muscle contraction-related genes in Ahnak KO kidney were analyzed through real-time quantitative polymerase chain reaction (qPCR). The gene expression levels of *Best3*, *Cacnb4*, *P2rx3* and *c-kit* were significantly downregulated in Ahnak KO mice compared to WT mice (Fig. 4I-M). The protein level was confirmed by western blotting (Fig. 4N). Calcium signaling regulator Best3 protein level was significantly reduced in Ahnak KO mouse Kidney at PN1.

### Analysis of ureter defects in Ahnak KO mice at PN1

**Fig. 5:**
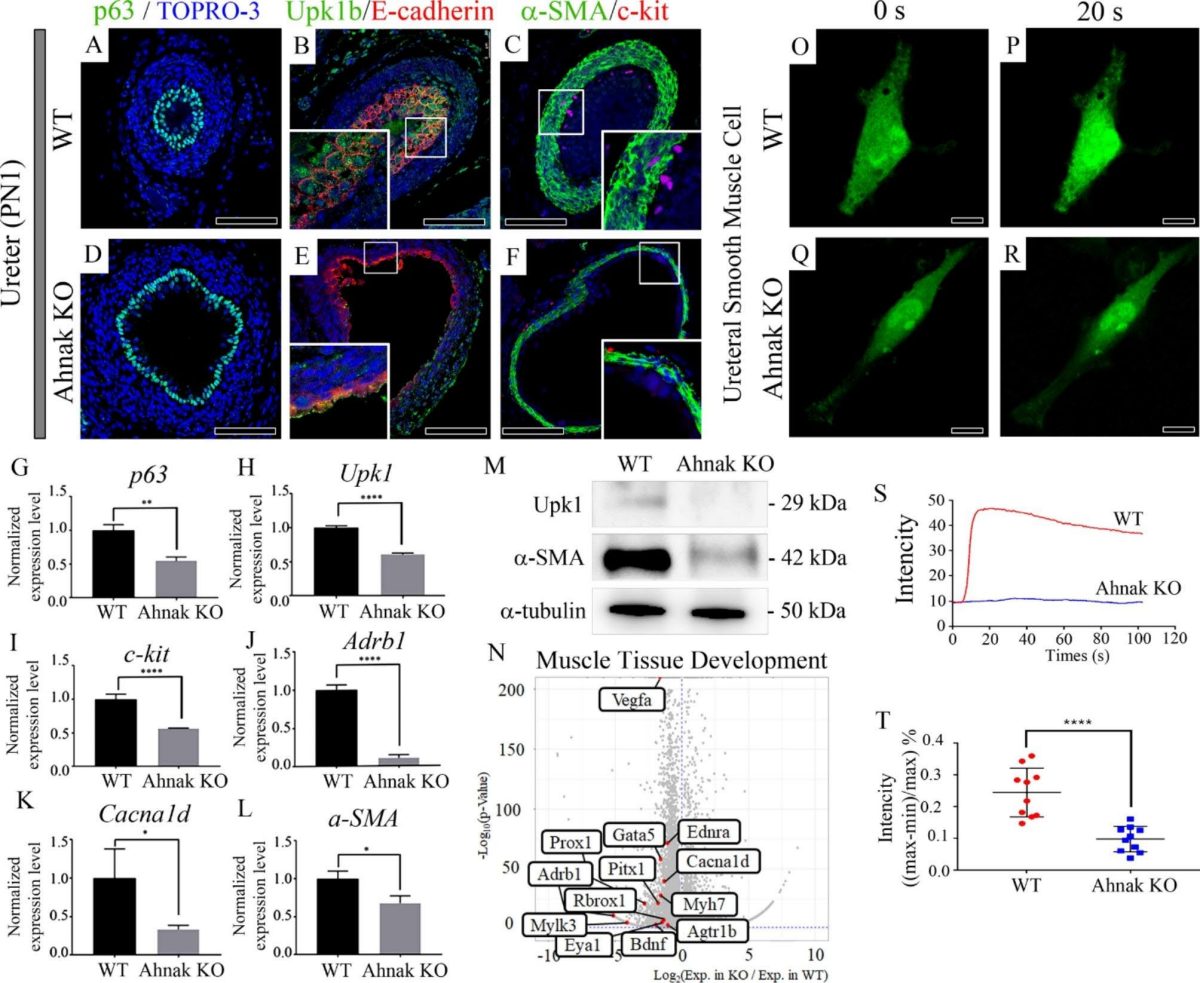
Alteration of ureter markers and calcium imaging in Ahnak KO mice at PN1. (**A**) p63 marks basal cell layer and multilayered basal cell layer is detected in WT ureter. (**D**) In Ahnak KO ureter, p63 positive mono basal cell layer and expanded lumen is observed at PN1. (**B**) Uroplakin 1b (Upk1b), a urothelium marker, and E-cadherin double-staining results show that multilayered urothelium is developed at PN1 in WT. (**E**) Upk1b and E-cadherin are weakly localized in Ahnak KO at PN1. (**C**) Thick α-SMA-positive muscularis layer is observed in WT ureter at PN1. c-kit, interstitial cells of Cajal-like cells (ICC-LCs) marker, is localized adjacent to the smooth muscle layer in WT ureter. (**F**) c-kit and α-SMA are weakly or not localized around thin smooth muscle layer in Ahnak KO mice. (**G**) Expression level of *p63* is reduced in Ahnak KO ureter compared to WT at PN1. (**H**) *Upk1b* expression level is decreased in Ahnak KO ureter compared with WT. (**I**) Relative expression level of *c-kit* is down-regulated in Ahnak KO ureter compared to WT at PN1. (**J**) *Adrb1* expression level is reduced in Ahank KO compared to WT. (**K**) Expression level of *Cacna1d* is decreased in Ahank KO ureter compared with WT. (**L**) α-SMA expression level is lower in Ahnak KO ureter compared to WT ureter at PN1. (**M**) Upk1b and α-SMA protein level is down-regulated in Ahnak KO mouse ureter at PN1. (**N**) Volcano plot presenting down-regulated genes related to muscle tissue development, such as *Adrb1* and *Cacna1d* in Ahnak KO ureter. (**O, P**) The green fluorescent protein (GFP) response by Fluo-4 is significantly increased in WT ureter smooth muscle cells after CaCl_2_ treatment. (**Q, R**) GFP response by Fluo-4 is not altered in Ahnak KO ureter smooth muscle cell after CaCl_2_ treatment. (**S**) GFP intensity is dramatically up-regulated in WT but not Ahnak KO ureter smooth muscle cells until 100 s after CaCl_2_ treatment. (**T**) GFP intensity of ureter smooth muscle cells ((max-min)/max) is much higher in WT compared to Ahnak KO (n = 10). *, *P* < 0.05; **, *P* < 0.01; ****, *P* < 0.0001; ns, not significant; Scale bar, A-F, 100 μm, O-R, 20 μm

Localization of p63, Upk1b, and c-kit, which are markers for basal cells, superficial cells, and peristalsis pacemakers, respectively, was examined in WT and Ahnak KO ureters at PN1 (Fig. 5A-F). Significant localization of p63 was detected in multi-layered basal cells in WT animals at PN1 (Fig. 5A). In the Ahnak KO ureter, p63 was observed in monolayer basal cells at PN1 (Fig. 5D). Upk1b was detected in the superficial cell layer of the urothelium, and E-cadherin was co-localized in the multi-layered urothelium in WT mice at PN1 (Fig. 5B). Upk1b was not significantly localized in the superficial cell layer, and E-cadherin was localized in the monolayer urothelium in Ahnak KO mice at PN1 (Fig. 5E). c-kit was detected in interstitial cells of Cajal–like cells (ICC-LCs) located in the well-developed α-SMA-positive ureter muscle layer in WT mice (Fig. 5C). c-kit positive cells were reduced in the adjacent α-SMA positive thin smooth muscle layer in the Ahnak KO ureter at PN1 (Fig. 5F).

To evaluate gene expression levels, qPCR of *p63, upk1b* and *c-kit* was performed at PN1 ureter. The expression levels of *p63* (*P* < 0.05), *upk1b* (*P* < 0.0001), and c-kit (*P* < 0.05) were reduced in Ahnak KO ureters compared with WT ureters at PN1 (Fig. G-I). To confirm calcium homeostasis and smooth muscle development, the expression levels of *Adrb1, Cacna1d* and *α-SMA* were determined by qPCR (Fig. 5J-L). The expression levels of calcium homeostasis regulator genes *Adrb1 and Cacna1d* were reduced in Ahnak KO mice (Fig. 5J,K). The expression level of α-SMA (*P* < 0.05) was reduced in Ahnak KO ureters compared with WT ureters (Fig. 5L). To confirm the protein level, western blotting was performed (Fig. 5M). Upk1b and α-SMA protein level was reduced in Ahnak KO mouse ureter at PN1. Decreased genes expression of muscle tissue development related genes in Ahnak KO was observed in the volcano plot compared with WT in the PN1 ureter (Fig. 5N). The 13 well known muscle tissue development related genes downregulated differentially expressed genes (DEGs), including calcium signaling regulators such as *Cacnb4Adrb1, Cana1d, Vegfa, Ednra, etc.*, were presented in a volcano plot for Ahnak KO ureter (Fig. 5N; DEGseq *P* < 0.05 and fold change > 2).

To examine the functional response of smooth muscle cells (SMCs), calcium imaging was performed on primary ureteral SMCs using Fluo-4 AM calcium indicator dye (Fig. 5O-T). The cytosolic calcium activation response was clearly observed in WT but not in Ahnak KO ureteral SMCs (Fig. 5O-R, Additional file 1: Movie S1, S2). The intensity of Fluo-4 AM was recorded in ureteral SMCs in both WT and Ahnak KO mice (Fig. 5S). After CaCl_2_ treatment, Fluo-4 AM intensity was remarkably increased in WT ureteral SMCs. However, Ahnak KO ureteral SMCs responded weakly to CaCl_2_ treatment (Fig. 5T).

## Discussion

Ahnak expression was previously identified using single-cell RNA sequencing in the kidney, especially in podocytes [17]. However, its localization and functions have not yet been elucidated in the renal system. In this study, we performed IHC and confirmed that Ahnak protein is broadly localized in the kidney and ureter. In addition, Ahnak was selectively localized in podocytes in the glomerulus and collecting duct membrane. Moreover, Ahnak was strongly observed in the Tm, TM, and TA during ureteral development (Fig. 1). From these results, we hypothesized that malfunction of kidney and ureter may have induced in Ahnak KO mice.

To confirm the function of Ahnak in kidney and ureter development, Ahnak KO mice were analyzed at E15.5 and PN1. Hydronephrosis and hydroureter formation were observed in Ahnak KO mice indicating that Ahnak may play pivotal roles during renal system development, especially for proper kidney and ureter formation (Fig. 2). To identify the specific role of Ahnak, bulk-RNA sequencing was performed on the kidneys and ureters of WT and Ahnak KO mice at PN1. RNA sequencing results indicated that Ahnak regulates cellular calcium homeostasis and voltage-gated ion channel activity in the kidney, cellular calcium homeostasis, muscle development, and muscle contraction in the ureter (Fig. 3).

Intensity of calcium regulator proteins Best3, Cacnb4, and P2rx3, were lower in Ahnak KO kidneys than in WT kidneys (Fig. 4). Best3 is a chloride channel activated by Ca^2+^ and contributes to smooth muscle contraction [18]. Cacnb4, detected in the cerebellum, kidney, and testis contributes to the function of calcium channels and regulates cell proliferation and cell cycle progression [19, 20]. P2rx3 is activated by ATP and is capable of admitting Ca^2+^ into the cytoplasm [21]. qPCR results revealed downregulated *Best3*, *Cacnb4* and *P2rx3* expression levels in Ahnak KO kidneys compared to WT, similar to the RNA-sequencing results (Fig. 4). In Ahnak KO mice, the levels of calcium-related genes were downregulated in the kidneys. Moreover, c-kit, which produces a peristaltic wave, was selectively reduced by Ahnak loss in the kidney pelvis. These results suggest that the loss of Ahnak in the kidney causes calcium imbalance and kidney diseases such as hydronephrosis.

Urothelium thickness was significantly reduced, as confirmed by p63 and Upk1 localization in Ahnak KO ureters. In addition, a thin smooth muscle layer and weak peristalsis trigger protein, c-kit, were observed in Ahnak KO mice compared to those in WT mice. qPCR results indicated that the expression levels of cellular calcium iron homeostasis, peristalsis trigger, and smooth muscle-related genes were reduced in Ahnak KO ureters compared to WT ureters (Fig. 5). These results indicate that defects in the ureter and hydroureter may be caused by calcium imbalance, peristalsis, and smooth muscle formation in Ahnak KO mice.

Peristalsis is one of most pivotal functions in ureter for discharge of urine from kidney to urinary bladder [22]. Peristalsis trigger, c-kit, was reduced in Ahnak KO mouse kidney and ureter. To confirm the contractive function of SMCs in the ureter, calcium imaging was performed on SMCs isolated from the proximal region of the ureter. After CaCl_2_ treatment, SMCs from Ahnak KO ureters did not respond to calcium (Fig. 5). These results showed that both peristalsis and muscle contraction function were absent in the SMCs of the ureters of Ahnak KO mice.

Previous studies have reported that non-obstructive hydronephrosis is caused by an excessive volume of urine [23],[24]. Ahnak KO newborn mice are generally eaten by female mice, which may be due to abnormalities. Therefore, we analyzed PN1 mice and could not have analyzed not only urine volume but also urine components.

## Conclusions

**Fig. 6:**
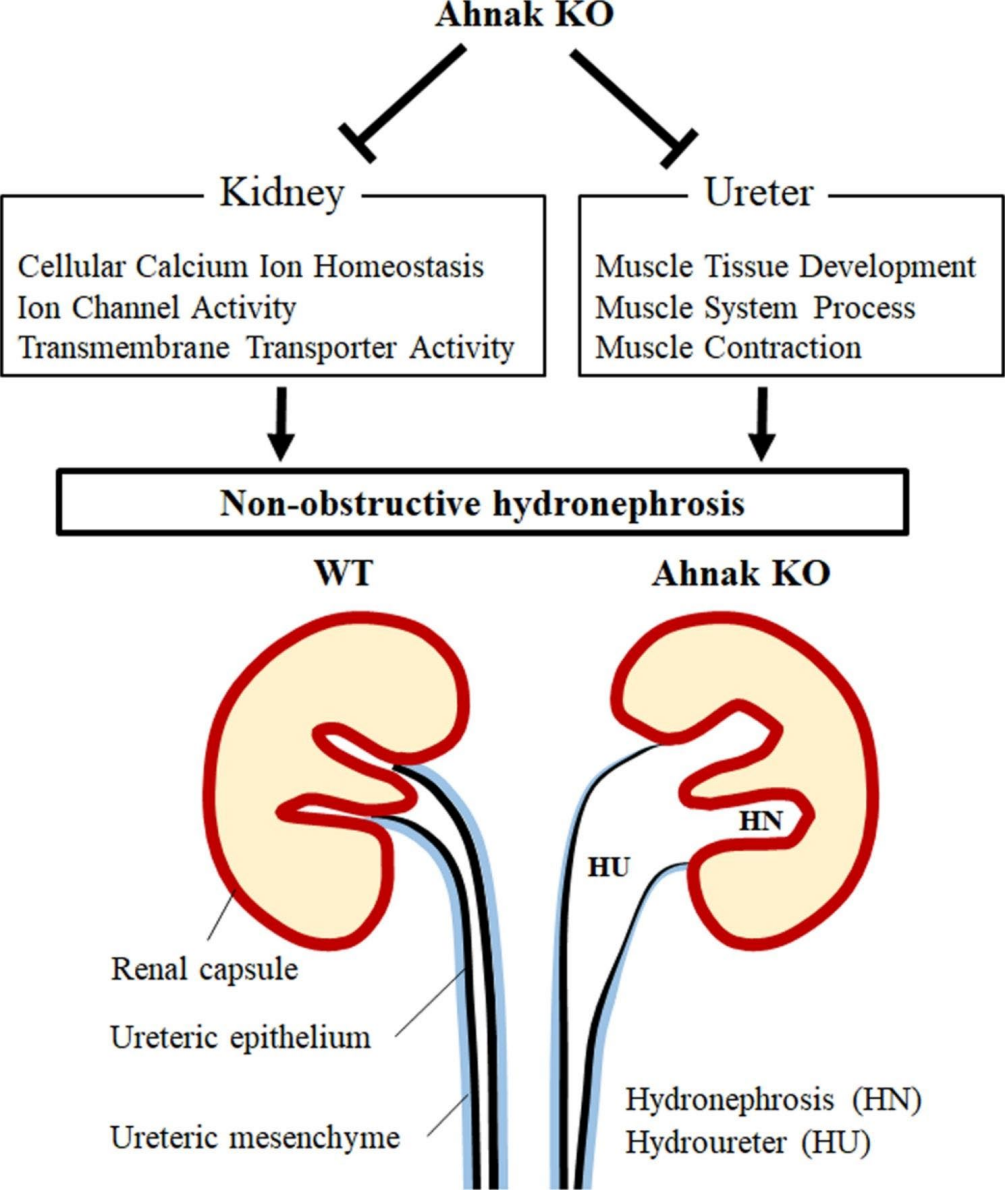
Schematic diagram of Ahnak KO in urinary system. The levels of ion channel activity, transmembrane transporter activity, and calcium ion homeostasis-related genes is reduced in Ahnak KO kidneys. Muscle development, muscle system processes, and muscle contraction-related gene expression is downregulated in the Ahnak KO ureter. This altered gene expression may induce hydronephrosis and hydroureter formation through the loss of calcium homeostasis and muscle development, including peristalsis

Ahnak is broadly localized in the kidney and ureter during development and regulates critical signaling in terms of ion channel activity, transmembrane transporter activity, cellular calcium ion homeostasis in the kidney, and muscle development, muscle contraction, and calcium ion homeostasis in the ureter. Moreover, hydronephrosis and hydroureter formation were observed in Ahnak KO mice, indicating that Ahnak plays a pivotal role in proper renal system formation by regulating calcium homeostasis and smooth muscle function (Fig. 6).

## Materials and methods

### Animals and animal maintenance

All animal experiments were approved by the Yonsei University Health System Institutional Animal Care and Use Committee (YUHS-IACUC) in accordance with the Guide for the Care and Use of Laboratory Animals (National Research Council, USA). The animal study plan for these experiments (2021-0096) was reviewed and approved by this committee. All experiments were performed in accordance with the guidelines of this committee.

Ahnak KO mice were kindly provided by Prof. Bae (Department of Life Sciences, Ewha Woman’s University, Seoul, Korea). Exon 5 of the Ahnak gene was disrupted to produce Ahnak KO mice, as reported previously. Using the previously reported procedures, genomic DNA extracted from tails was used for genotyping [25]. In this study, embryonic day (E) 12.5 (Mesonephros), E15.5 (Metanephros), and postnatal day (PN) 1 immediately after birth, which were reported to be important stage of renal development in previous studies, were analyzed [26]. Embryos were dissected renal system and analyzed WT and Ahnak KO at E12.5, E15.5 and PN1 in PBS. Specimens were fixed in 4% PFA/PBS.

### Bulk RNA-sequencing (RNA-seq)

2–4 kidney or ureter from PN1 mice were obtained and RNA was extracted by TRIzol. mRNA libraries were prepared using TruSeq Stranded mRNA Preparation kit (Illumina, CA, USA) according to the manufacturer’s instruction. RNA-seq was performed using Illumina HiSeq2500 sequencing platform. Cutadapt (version 2.8) was used to trim adaptor sequence and discard low quality reads [27]. Two biological replicates for each species were performed with RNA-seq, and were analyzed. The trimmed and filtered reads were mapped on reference genome (GRCM Reference 39, INSDC Assembly GCA_000001635.9) using STAR version 2.7.1a [28]. The expression level of genes and transcripts were calculated using Cufflinks version 2.2.1 [29]. Differentially expressed genes (DEGs) were identified using DESeq version 1.44.0 (Anders and Huber 2010) with p-Value < 0.05 and |fold change| > 2 threshold. Gene set analysis of significant DEGs for gene ontology (GO) terms were performed using clusterProfiler (version 4.0.4), a R package for interpretation of omics data [30]. Visaulization of DEGs on a map of signaling pathway was performed using Pathview version 1.30.1, a R package for pathway based data integration and visualization [31]. The RNA-seq data have been deposited in the Gene Express Omnibus (GEO) database [GEO: GSE218925]. The statistical analysis and visualization of RNA-seq data was performed by NGeneS Inc. (Ansan-si, Republic of Korea).

### Morphology analysis on kidney and ureter of Ahnak knockout animal

The statistical analysis was performed for confirm kidney and ureter phenotype difference with WT and Ahnak KO. Analyzing factor is kidney width, hydronephrosis kidney ratio, hydroureter ratio and urothelium thickness. To analyze factor in kidney and ureter, software (Image J) was used. Kidney is measured the length from pelvic to outline of kidney for obtaining kidney width. Urothelium is measured the length from tip of superficial cell layer to basal cell, located in inner smooth muscle for obtaining urothelium thickness. Count number of hydronephrotic kidney among WT and Ahnak KO kidney. Count number of hydroureter among WT and Ahnak KO ureter. Calculating ratio of hydronephrosis/total kidney and hydroureter/total ureter in WT and Ahnak KO for each.

### Histology and immunofluorescence

The samples were fixed in 4% paraformaldehyde and processed as per the standard procedure. Six-micron-thick sections were prepared for hematoxylin/eosin (H&E) staining and immunostaining. The specimens were boiled in citrate buffer (pH 6.0) for antigen retrieval and blocked using 1% goat serum or 5% bovine serum albumin in PBS. The specimens were incubated with primary antibodies against monoclonal rabbit anti-Ahnak (1:200, produced by Prof. Bae’s lab, Department of Life Sciences, Ewha Woman’s University, Seoul, Korea), monoclonal rabbit anti-p63 (1:200, GeneTex), monoclonal rabbit anti-upk1b (1:200, Invitrogen), monoclonal mouse anti-E-cadherin (1:200, BD Transduction), monoclonal rabbit anti-c-kit (1:100, Abcam), monoclonal mouse anti-α-SMA (1:200, Invitrogen) at 4 °C overnight. The following day, these sections were incubated with a secondary antibody Alexa488-conjugated goat-anti-rabbit IgG (1:200, Invitrogen), Alexa488-conjugated goat-anti-mouse IgG (1:200, Invitrogen), Alexa555-conjugated goat-anti-mouse IgG (1:200, Invitrogen), Alexa555-conjugated goat-anti-rabbit IgG (1:200, Invitrogen) and counterstained with TO-PROTM-3 (T3605, Invitrogen, OR, USA; 1:1000) to visualize nuclei. All specimens were examined using a confocal laser microscope (DMi8, Leica, Germany).

### Real-time quantitative polymerase chain reaction (qPCR)

qPCR was performed on extracted DNA from PN1 kidney and ureter in WT and Ahnak KO. Total RNA was extracted from kidney and ureter using TRIzol® reagent (#15596-026, Thermo Fisher Scientific, USA). cDNA was made from the mRNA of each type of tissue using Maxime RT PreMix according to manufacturer’s instructions (#25,081, iNtRON, Korea). qPCR was performed using a StepOnePlus Real-Time PCR System (Applied BioSystems, USA). qPCR was performed using a StepOnePlus Real-Time PCR System (Applied Biosystems). The amplification program consisted of 40 cycles of denaturation for 15 s, annealing for 1 min and extension for 20 s at primer specific temperature. The expression levels of each gene are expressed as normalized ratios against the β2 microglobulin (B2M) housekeeping gene. Primers sequence for pPCR in selected genes were shown in Additional file 1: Table S1.

### Western blot

Cell extracts from PN1 kidney or ureter tissues were fractionated by SDS-PAGE and transferred to a polyvinylidene difluoride membrane (PVDF; Millipore) using a transfer apparatus according to the manufacturer’s protocols. After incubation with 5% skim milk in TBST (10 mM Tris, pH 7.4, 150 mM NaCl, 0.1% Tween 20) for 1 h, the membrane was incubated with primary antibodies at 4 °C overnight. Membranes were washed three times for 10 min and incubated with HRP-conjugated secondary antibodies for 2 h. Blots were washed three times with TBST and developed with the ECL system (RPN2232, GE Healthcare Life Sciences, USA) according to the manufacturer’s protocols.

### Statistical analysis

The graphic results were expressed as the mean ± standard deviation (SD). A GraphPad Prism 7 (GraphPad Software, San Diego, CA, USA) was used to analyze the data. Comparison of multiple groups was performed by one-way ANOVA followed by Tukey’s multiple comparisons test. A p value < 0.05 was considered significant.

## Electronic supplementary material

Below is the link to the electronic supplementary material.

Supplementary Material 1

Supplementary Material 2

Supplementary Material 3

## Data Availability

The RNA-seq data have been deposited in the Gene Express Omnibus (GEO) database [GEO: GSE218925].

## Abbreviations

CAKUT: congenital anomalies of the kidney and urinary tract
KO: knockout
IHC: immunohistochemistry
UB: ureteric bud
MM: metanephric mesenchyme
K8: Keratin-8
WT1: Wilms tumor-1
LTL: Lotus Tetragonolobus Lectin
TA: tunica adventitia
TM: tunica muscularis
Tm: tunica mucosa
H&E: hematoxylin and eosin
WT: wild-type
PN: postnatal
DEGs: differentially expressed genes
GO: Gene Ontology
qPCR: real-time quantitative polymerase chain reaction
RNA-seq: Bulk RNA-sequencing
GEO: Gene Express Omnibus
