# Supplementary material for: Ahnak is required to balance calcium ion homeostasis and smooth muscle development in the urinary system
Source: Cell Biosci. 2023 Jun 12;13:108. doi: 10.1186/s13578-023-01055-x (PMC10262403; doi:10.1186/s13578-023-01055-x)
Supplement: Supplementary file 1 — Supplementary Material 1 [file 13578_2023_1055_MOESM1_ESM.docx]

**Table S1.** Target primer sequence for qPCR

|  | **Target primer** | **Forward** |
| --- | --- | --- |
| 1 | Best3 | Forward : 5’ – CCA GTA ACC TTT GTG CTT GGG - 3’ |
|  |  | Reverse : 5’- CAT CAG CGT CCT TCT GAG CA - 3’ |
| 2 | Cacnb4 | Forward : 5’- GAG GGC TGT GAG ATT GGC TT -3’ |
|  |  | Reverse : 5’- TGT GGG AGT TGC TCG GAA TG - 3’ |
| 3 | P2rx3 | Forward : 5’- CCT ACT TTG TGG GGT GGG TT -3’ |
|  |  | Reverse : 5’- CTG TTG GCA TAG CGT CCG AA - 3’ |
| 4 | c-kit | Forward : 5’- ATA GAC CCG ACG CAA CTT CC -3’ |
|  |  | Reverse : 5’- GCA TCT TCA CGG CAA CTG TC - 3’ |
| 5 | α-SMA | Forward : 5’- CAC GAA ACC ACC TAT AAC AGC ATC -3’ |
|  |  | Reverse : 5’- CCA GAC AGA GTA CTT GCG TTC T - 3’ |
| 6 | p63 | Forward : 5’- ATG ATG GGC ACT CAC ATG CC -3’ |
|  |  | Reverse : 5’- GAG CAG CCC AAC CTT GCT AA - 3’ |
| 7 | upk1b | Forward : 5’- CAG CAA CAC AAC GCG ACT TT -3’ |
|  |  | Reverse : 5’- GTC ATT GGT TGG GGG ACT GT - 3’ |
| 8 | Adrb1 | Forward : 5’- CTG CTA CAA CGA CCC CAA GT -3’ |
|  |  | Reverse : 5’- CAC GTA GAA GGA GAC GAC GG - 3’ |
| 9 | Cacna1d | Forward : 5’- TTA GTG ACG CCT GGA ACA CG -3’ |
|  |  | Reverse : 5’- TTT CAG ATG GGT CGG CTT CG - 3’ |
| 10 | B2m | Forward : 5’- CCT GGT CTT TCT GGT GCT TG -3’ |
|  |  | Reverse : 5’- CCG TTC TTC AGC ATT TGG AT - 3’ |

**Table S2.** Top 15 down-regulated genes in Ahnak KO Kidney

|  | **DEG** | **Description** | **log2.Fold_change** | **p.value** |
| --- | --- | --- | --- | --- |
| 1 | Grin2c | glutamate receptor, ionotropic, NMDA2C (epsilon 3) | -2.784723795 | 0.021526006 |
| 2 | Cacnb4 | calcium channel, voltage-dependent, beta 4 subunit | -2.318066771 | 3.85E-201 |
| 3 | Kcng4 | potassium voltage-gated channel, subfamily G, member 4 | -3.954648796 | 6.39E-05 |
| 4 | Kcnh4 | potassium voltage-gated channel, subfamily H (eag-related), member 4 | -3.485163513 | 0.001161971 |
| 5 | Scn3b | sodium channel, voltage-gated, type III, beta | -3.244155413 | 0.003734321 |
| 6 | Best3 | bestrophin 3 | -4.308285751 | 3.64E-06 |
| 7 | Ankmy1 | ankyrin repeat and MYND domain containing 1 | -4.177041217 | 1.14E-05 |
| 8 | Gabrd | gamma-aminobutyric acid (GABA) A receptor, subunit delta | -3.954648796 | 6.39E-05 |
| 9 | Grid2 | glutamate receptor, ionotropic, delta 2 | -3.244155413 | 0.003734321 |
| 10 | Best1 | bestrophin 1 | -1.124799236 | 6.04E-10 |
| 11 | P2rx3 | purinergic receptor P2X, ligand-gated ion channel, 3 | -5.829117914 | 3.50E-16 |
| 12 | Pkd2l2 | polycystic kidney disease 2-like 2 | -4.485163513 | 6.66E-07 |
| 13 | Oxt | oxytocin | -3.485163513 | 0.001161971 |
| 14 | Ghrl | ghrelin | -3.369686295 | 0.00208244 |
| 15 | Aqp5 | aquaporin 5 | -1.249846525 | 8.18E-08 |

**Table S3.** Top 15 down-regulated genes in Ahnak KO Ureter

|  | **DEG** | **Description** | **log2.Fold_change** | **p-value** |
| --- | --- | --- | --- | --- |
| 1 | Adrb1 | adrenergic receptor, beta 1 | -5.137462579 | 6.65E-12 |
| 2 | Actc1 | actin, alpha, cardiac muscle 1 | -2.225776051 | 0 |
| 3 | Bmp10 | bone morphogenetic protein 10 | -4.164429626 | 2.58E-06 |
| 4 | Myl2 | myosin, light polypeptide 2, regulatory, cardiac, slow | -4.538825141 | 4.57E-08 |
| 5 | Myoz1 | myozenin 1 | -5.835806879 | 1.35E-18 |
| 6 | Myoz2 | myozenin 2 | -5.267523119 | 6.05E-13 |
| 7 | Acta1 | actin, alpha 1, skeletal muscle | -1.505113544 | 3.96E-187 |
| 8 | Cacna1d | calcium channel, voltage-dependent, L type, alpha 1D subunit | -1.320920111 | 1.60E-40 |
| 9 | Cacna1h | calcium channel, voltage-dependent, T type, alpha 1H subunit | -1.081967466 | 4.96E-59 |
| 10 | Kcnq1 | potassium voltage-gated channel, subfamily Q, member 1 | -5.713322872 | 3.29E-17 |
| 11 | Scn5a | sodium channel, voltage-gated, type V, alpha | -5.749392127 | 1.32E-17 |
| 12 | Neb | nebulin | -3.109981842 | 5.36E-26 |
| 13 | Nrg1 | neuregulin 1 | -5.431909937 | 2.20E-14 |
| 14 | Oxtr | oxytocin receptor | -5.024251968 | 4.60E-11 |
| 15 | Adgrb1 | adrenergic receptor, beta 1 | -5.137462579 | 6.65E-12 |

**Movie S1**

GFP response by Fluo-4 is significantly increased in WT ureter smooth muscle cells after CaCl_2_ treatment.

**Movie S2**After CaCl_2_ treatment, GFP response by Fluo-4 is not altered in Ahnak KO ureter smooth muscle cell.
